# Music therapy with adults in the subacute phase after stroke: A study protocol

**DOI:** 10.1016/j.conctc.2024.101340

**Published:** 2024-07-25

**Authors:** Theo Dimitriadis, Mohammed A. Mudarris, Dieuwke S. Veldhuijzen, Andrea W.M. Evers, Wendy L. Magee, Rebecca S. Schaefer

**Affiliations:** a Health, Medical, and Neuropsychology Unit, Institute of Psychology, Faculty of Social and Behavioural Sciences, Leiden University, P.O. Box 9555, 2300RB, Leiden, the Netherlands; b Amstelring Rehabilitation Centre and Nursing Homes, Saaftingestraat 8, 1069BW, Amsterdam, the Netherlands; c University of Jeddah, College of Science and Arts at AlKamil, Department of Communication Skills, Jeddah, Saudi Arabia; d Leiden Institute for Brain and Cognition, Leiden University, the Netherlands; e Medical Delta Healthy Society, Leiden University, Technical University Delft and Erasmus University Rotterdam, the Netherlands; f Boyer College of Music and Dance, Temple University, Philadelphia, PA, USA; g Academy of Creative and Performing Arts, Leiden University, the Netherlands

**Keywords:** Music therapy, Subacute stroke, Motivation, Motor rehabilitation, Cognitive rehabilitation

## Abstract

Stroke is a highly disabling condition, for which music therapy is regularly used in rehabilitation. One possible mechanism for the effects of music therapy is the motivational aspect of music, for example regarding treatment adherence based on improved mood. In this study, changes in motivation related to Neurologic Music Therapy (NMT) techniques during rehabilitation in the subacute phase after stroke will be investigated. Using a randomised within-subjects cross-over design, the effects of two NMT techniques and related motivational indices will be investigated. Data will be collected at three timepoints: baseline (TP1), after being randomised into groups and receiving NMT or standard care (TP2), and then at a third time point after the cross-over and having received both conditions (TP3). This design allows to counteract order effects, time effects due to spontaneous and/or nonlinear recovery, as well as single-subject comparisons in a relatively heterogeneous sample. Twenty adult participants who have experienced a supratentorial ischaemic or haemorrhagic stroke and are experiencing upper-limb impairments and/or cognitive deficits will be included. Behavioural measures of motor function, cognition, and quality of life will be collected, along with self-reported indices of overall motivation. The study outcomes will have implications for the understanding of the underlying mechanisms of music therapy in stroke recovery, more specifically regarding the relevance of motivational states in neurorehabilitation.

## 1 Introduction

Music-based interventions have been used successfully in the treatment of impairments after stroke and have been shown to improve various functions, such as gait [1], speech/language [2], impaired arm motor function [[3], [4]], and attention [5]. Possible underlying mechanisms have been described as focusing on increased repetition, potentially improved temporal skills, motivation, and multisensory embedding of movement [[6], [7]]. Despite these aspects of music being commonly used by music therapists in their daily clinical practice [8] and Music Therapy being regularly offered as part of a rehabilitation program, there remains a gap in the scientific evidence to support these methods, and the empirical support for its underlying mechanisms. Specifically, motivational aspects of music, arguably due to improved mood and emotion regulation affecting the drive to improve, have been found to affect clinical and nonclinical outcomes in a systematic review [9]. In the current paper, we conceptualise motivation as the drive to participate in therapy and self-regulate emotions. We also focus on techniques drawn from Neurologic Music Therapy (NMT), a clinical system of music interventions based on the neuroscience of music perception, cognition, and production and how music can influence and change non-musical brain and behaviour function.

Neurological and functional recovery (e.g. movement, cognition and/or independent living) primarily occurs in the acute and sub-acute phase [10], and only 5–20 % of patients ever fully recover in terms of their upper-limb function [11], including arm and hand movements. Cognitive deficits occur in more than half of stroke survivors, with attentional deficits as the most prominent. Attention is linked to motor recovery, as it is considered essential for motor learning [12]. The interaction between movement and cognition is commonly seen in people who have experienced a stroke through combined presentation of symptoms and related recovery. For instance, the level of sustained attention at two months post-stroke was found to predict motor recovery two years later [13], and music-based interventions have shown potential to improve both domains (e.g. Refs. [[14], [15]]).

In the field of neurorehabilitation, motivation has been demonstrated to be a link between cognition and motor performance, playing an important role in rehabilitation outcome determining factors [16]. Motivation is therefore considered to be a mechanism for functional improvements (e.g. Ref. [13]), including in music therapy, likely due to potential mood improvements [14], but it has not been explicitly investigated [9]. Moreover, in previous research, motivational differences have anecdotally been viewed as confounders, rather than an important aspect of the therapeutic process that may also affect elements of a patient's functional level. The motivational potential of music is well-established in athletics [17], while the effect of music on motivation is more often formulated in terms of mood, which is more generally extensively investigated and utilised in clinical settings to regulate stress and anxiety (e.g. Ref. [18]). Physiological correlates of stress reduction with music more generally are increasingly elucidated [19] with potential for application in many other settings. In further support of the role of motivation in music-based intervention methods, studies have also found that movement rehabilitation utilising music may be more effective than those without an auditory component [1,20] with evidence supporting improvements in motor function, mood, and cognition of patients [21]. Interestingly, personality traits, such as agreeableness, extroversion, openness, neuroticism and conscientiousness, have been linked to not only music-based movement [22] and musical emotion regulation [23], but also well-being measures during stroke recovery, including quality of life [24], and mood [25]. While possibilities for quantifying motivational states are still in the early stages, validated measures have been developed focusing specifically on motivation for rehabilitation in stroke [26].

In the current study, we aim to assess variations in the intrinsic motivation to engage in musical activities over three timepoints: at baseline (TP1), after either the intervention or control condition (TP2), and after the other condition (TP3) in a cross-over design. Studies in patients with mood disorders have shown that engaging in music therapy was associated with adherence to other therapeutic interventions, such as physical therapy or occupational therapy [27,28]. While this has not been demonstrated in stroke patients, we seek to examine if music therapy is associated with higher attendance to other therapies. Research examining both intrinsic and extrinsic motivation has focused on music-reward in wanting versus liking paradigms [29], as an indication of motivation [30,31], but this has not been examined in stroke patients undergoing music therapy, for whom motivation is thought to underlie functional recovery [32].

The main aim of this project is to investigate within-subject changes in motivation during two NMT interventions. Secondly, we aim to assess the direct effects of the two NMT interventions on motoric and cognitive functional outcomes (in particular upper limb function and attention/concentration) and subjective well-being, as compared to a within-subject control period consisting of usual (or standard) care. Here, several caveats should be taken into account, that stem from not having an active control condition that would assess NMT-specific motivational effects rather than the effect of adding additional NMT to standard care. While there are aspects of usual care that preclude standardisation (see also [33]), more recent guidelines indicate that its content should primarily be driven by the trial's requirements to protect participants, inform practice, and be methodologically robust, efficient, feasible and acceptable to stakeholders [34]. Based on exchanges with the care facility and its multidisciplinary team, a standard care control period was deemed most pragmatic, as well as fitting for the target group and clinical context.

We hypothesise that NMT techniques targeting motor and cognitive outcomes will lead to increased self-reported overall motivation as compared to a period of no treatment. Secondly, we expect that changes in motivation will correlate with changes in functional outcomes in motor, cognitive, and mood domains. In terms of effectiveness, we expect the NMT interventions to positively affect functional outcomes within each participant. Finally, the study also aims to explore whether changes in motivation predict self-reported ratings of well-being, and whether participating in NMT increases adherence to other rehabilitation therapies. As a secondary aim, the effectiveness of the two NMT interventions will be assessed by comparing it to our care-as-usual waiting period. Here, we expect clinical outcomes of motor and cognitive function to improve more with additional NMT than without, controlling for the post-incident recovery time.

## 2 Protocol

### 2.1 Objectives

The primary objective is to investigate increases in motivation as a hypothesised mechanism underlying the effectiveness of two NMT interventions for stroke patients experiencing upper-limb impairments and/or cognitive deficits. The secondary objective is to assess the within-subject effects of the NMT interventions as compared to the control period on behavioural outcomes of motor functions, cognitive functions, and self-reported measures of well-being.

### 2.2 Study design

The study will employ a within-subject cross-over design; as stroke patients are not a homogenous group, this design is adopted to control for expected within-subject variation. To account for variability in brain damage that is caused by stroke, each subject thus serves as their own control. The cross-over design will account for order effects, as participants will be randomised into either receiving the intervention or the control phase first (AB), and vice versa (BA), while also allowing for the assessment of the intervention effectiveness (as part of the secondary goal).

All subjects will receive two NMT techniques targeting both upper-limb movement and attention. The study aims for participants to receive 9 sessions of NMT over 3–6 weeks, however, participants who complete a minimum of 6 sessions will be included in the primary analysis. The frequency of clinical contacts will aim for an average of 2–3 sessions per week but will be adjusted to a patient's expected length of stay and ability, using a tailored approach. Dosage will depend on a participant's rehabilitation trajectory, as subacute patients generally are admitted for a minimum of ±6 and a maximum of 16 weeks.

**Fig. 1:**
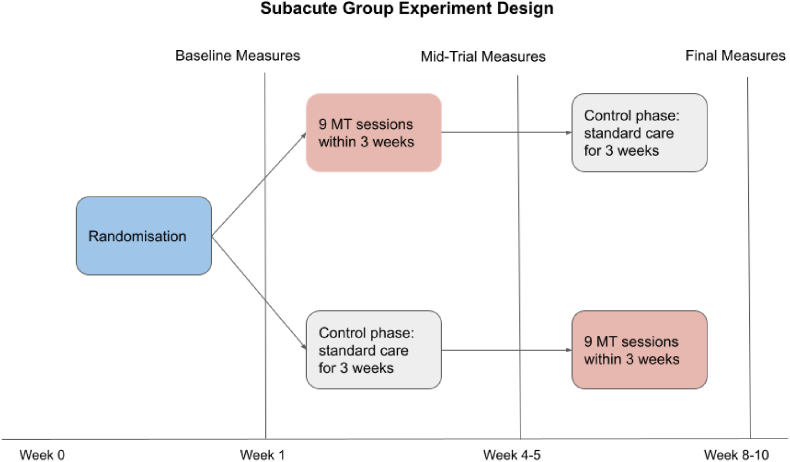
Experiment Design (example of a rehabilitation period of 8–10 weeks). *Note*: Timeline from inclusion to study completion, depicting the cross-over design and the most common range of durations of experimental and control periods.

Fig. 1 gives an overview of the design and procedures that the subjects will undergo within the course of the research, based on a rehabilitation trajectory of 8–10 weeks. When participants’ rehabilitation trajectory is shorter (6–8 weeks) or longer (12–16 weeks), then the intervals between baseline, mid-trial and final measures are adjusted accordingly.

### 2.3 Study population

Twenty adults (≥18 years of age) with supratentorial stroke admitted to the stroke unit of the Leo Polak Rehabilitation Centre in Amsterdam, will be recruited within the subacute phase (2 weeks - 6 months after the incident).

#### 2.3.1 Sample size calculation/power analysis

In a previous study, 40 stroke patients in the sub-acute phase were allocated to either 20 30-min sessions of music-supported therapy (N = 20; 4 withdrew/excluded; 16 in final analysis) or twenty additional sessions of conventional therapy (N = 20; 2 withdrew/excluded; 18 in final analysis) [35]. The study found a significant correlation (r = 0.562, p = 0.024) between patients’ intrinsic motivation to engage in musical activities as measured by the Barcelona Music Reward Questionnaire (BMRQ, [36]) at baseline and improvement of upper-limb function as measured by the Action Research Arm Test (ARAT, [37]) at 3 months, which was not found in the comparator of occupational/physical therapy (r = −0.305, p < 0.21), with a significant difference between correlations (z = 2.4, p < 0.05). Using Gpower 3 analysis, we estimated that 18 participants are needed to observe a similar effect size for a sufficiently powered study (α = 0.05 and (1-β) = 0.8; ρ H1 = 0.56). This indicates that 20 participants completing the entire protocol would be required to observe an effect of motivation in music therapy, with a hypothesised attrition/missing data rate of 10–20 %. This sample size is similar to the number of participants used in studies showing an effect for our secondary objectives, ranging from 14 to 20 participants, on the effects of music therapy on motor function [38], cognition, and psychological wellbeing [39]. Recruitment will continue until 20 participants have completed the protocol.

#### 2.3.2 Inclusion criteria

In order to be eligible to participate in the study, a participant should be above 18 years of age and have had their first-ever supratentorial stroke between 2 weeks and up to 6 months before enrolment. Moreover, participants will have a referral for a paretic upper limb, i.e., partial loss of the capacity to carry out a voluntary upper limb movement and/or difficulty with attention/concentration (e.g., complaints of being easily distractible, or unable to focus on a specific task in the presence of competing information, or decline in attention). Participants must be able to receive music therapy for a minimum of 2–3 sessions per week, aiming for a total of 9 sessions over a minimum of three weeks, reducing this to 6 sessions in 2 weeks if required. The frequency of sessions is based on the individual's ability and expected length of stay. Good command of the Dutch or English language is also a prerequisite for participation.

#### 2.3.3 Exclusion criteria

**Fig. 2:**
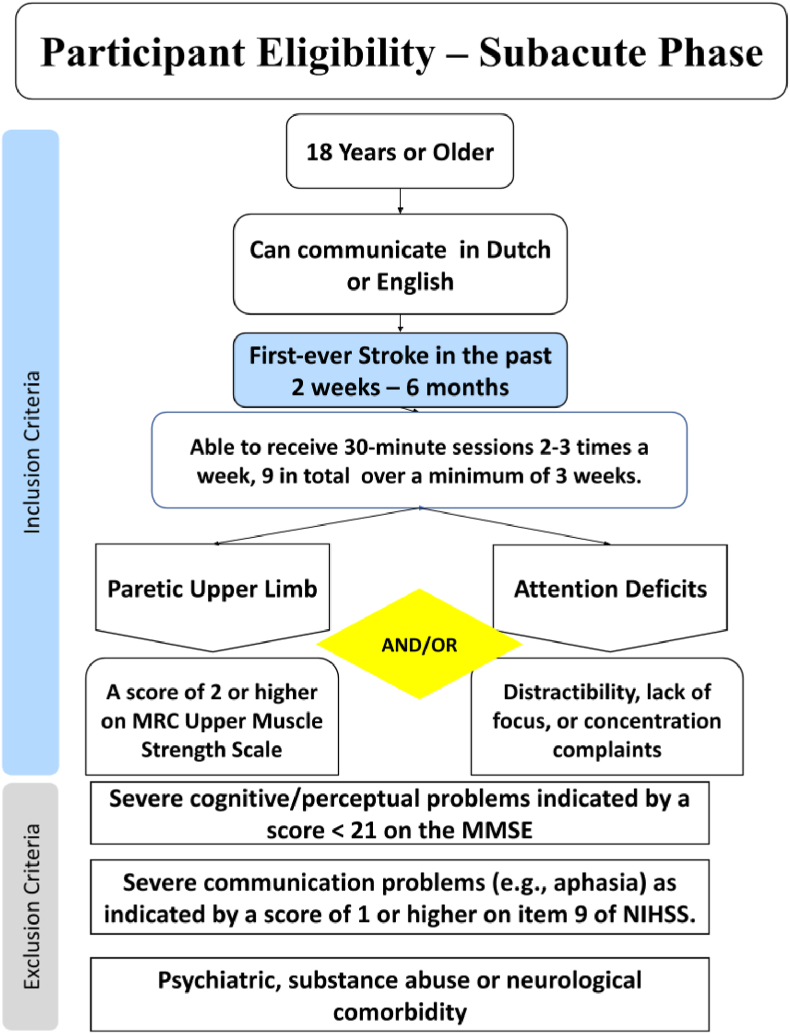
Participant eligibility.

Participants will be excluded if this is not their first stroke or if a second stroke occurs during their stay at the rehabilitation centre. Further exclusion criteria are the following: i) upper limb paralysis as assessed by a score <2 points on the Medical Research Scale (MRC) for muscle strength [40]; ii) severe behavioural and/or cognitive problems preventing understanding or carrying out instructions or risk of dementia (a score <21 on the Mini-mental state examination; MMSE; [41]); iii) severe communication problems, e.g., severe aphasia and/or apraxia of speech; iv) known psychiatric, substance abuse, or neurological comorbidity. For participants who score <24 on the MMSE, a follow-up check will be carried out for a possible dementia diagnosis which would retrospectively lead to exclusion. Fig. 2 illustrates the criteria for inclusion and exclusion for this study.

#### 2.3.4 Incentives

As a token of appreciation, participants will be provided a gift card of €20 for participating in the study.

### 2.4 Treatment of subjects

The following two protocolised NMT techniques targeting motor recovery and attention training [8], will be used: Therapeutic Instrumental Music Performance (TIMP) and Musical Attention Control Training (MACT). Depending on the rehabilitation goals, namely upper limb recovery, increase of attention/concentration, or both, one or both methods are utilised during a music therapy session.

Therapeutic Instrumental Music Performance (TIMP) is a technique that utilises musical instruments to help patients to exercise impaired motor function and regain functional patterns of movement. TIMP musical exercises are designed to target specific movement patterns (such as pinching, grasping or a gross motor arm movement), with maximum repetition. TIMP involves the planning of functional exercises using musical instrument playing to meet functional physical goals, set within the multidisciplinary team, with the aim of transferring the therapeutic learning into real-world applications. The tactile and auditory feedback from playing musical instruments as a part of a structured program of upper limb rehabilitation following stroke create so-called audio-motor coupling [42], thought to facilitate learning. During each session clients will perform clear rhythmical patterns using the affected upper limb, while the music therapist accompanies them with a harmonic musical instrument (e.g. piano or guitar) adjusted at the beginning of each session according to the state and progress of each client [43]. The duration of each NMT session will be 30 min. Musical Attention Control Training (MACT) is a technique that provides structured active or receptive musical exercises involving precomposed performance or improvisation, in which musical elements cue different musical responses, to practise specific attention functions, namely: sustained, selective attention, inhibition/switching, and turn-taking [44]. When linked with non-musical information, music adds structure and organisation, emotion, and appeal to the information in order to increase the probability that attention will be focused, maintained and/or switched [8]. The benefits of the neurologic music therapy protocols used in this study (TIMP and MACT) in particular for stroke survivors have been previously reported [[45], [46], [47]] although the evidence is limited and, arguably, not well established.

The Therapeutic Instrumental Music Performance (TIMP) exercises will focus on functional improvement of the affected upper limb: playing a percussion instrument through elbow extension, supination and protonation, wrist and finger flexion and extension. The Musical Attention Control Training (MACT) exercises will focus on the improvement of sustained, selective and divided attention through the use of one or more musical stimuli, for instance playing to the beat of a chosen song.

### 2.5 Methods

#### 2.5.1 Study parameters

The main parameters of the study focus on the differences in motivation levels of participants as the result of applying NMT techniques in the subacute phase of stroke, comparing within-subject differences between changes related to the intervention and the control period. The motivation level of participants will be measured using the Brain Injury Rehabilitation Trust Motivation Questionnaire BMQ-S [self-reported] [26] as state-level motivation. The BMQ-S is a 34-item questionnaire, which measures the motivation levels of the stroke survivor, using a 4-point Likert scale (always, often, sometimes, never). Items that involve different areas such as anhedonia, perseveration, poor initiation or distractibility, are summed to create a total score from 34 to 136, with higher scores representing greater difficulties in motivation. This measure will be administered at multiple time points, before and after the intervention and control periods (Baseline/TP1, Mid-trial/TP2 and Final Measures/TP3, see Fig. 1).

The secondary parameters relate to behavioural outcomes of upper limb, and cognitive functions, as well as self-reported measures of well-being following NMT for within-participant comparisons between the intervention and control period. The outcome measure of motor function that will be administered is the Action Research Arm Test (ARAT) [37] to assess grip, grasp, pinch and gross movement. Activities of daily living related to upper-limb function (including feeding, bathing, and dressing) will be measured by the Barthel Index [48].

Cognition will be assessed through the following outcome measures: Cancellation WAIS-IV (selective visual attention and perceptual speed) [49], Colour-Word Interference test (also known as the Stroop test) (sensitivity to interference) [50], Digit-span (attention span), verbal fluency (executive function and process speed), and the trail-making test (switching/divided attention).

**Fig. 3:**
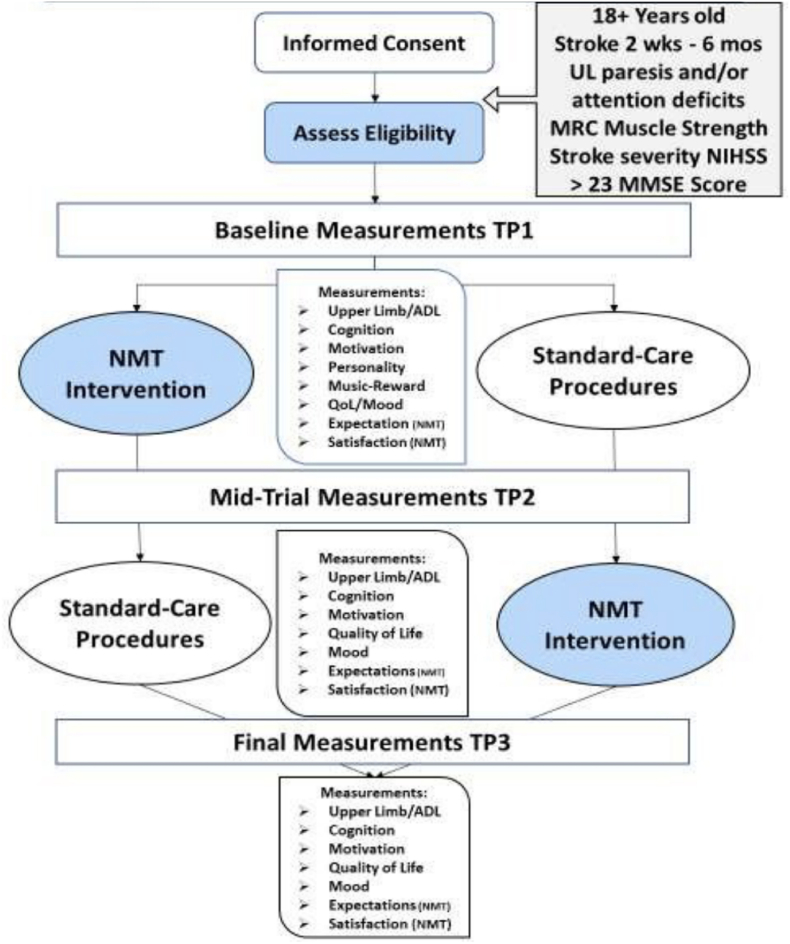
Study procedure.

In terms of changes of well-being pre- and post-intervention, together with self-rated motivation for other aspects of rehabilitation (e.g., physical therapy, speech therapy, occupational therapy, etc), quality of life will be measured by the EuroQol-5 (EQ-5D; [51]), and mood by Depression, Anxiety Stress Scale-21 checklist (DASS-21; [52]). Additionally, a Visual Analogue Mood Scale (VAMS, [53]) will be administered, pre/post each music therapy session, and trait questionnaires relating to personality by the Big Five Inventory (Extra Short Form) (BFI-2-XS; [54]) and optimism, as measured by the LOT-R [55], and The Barcelona Music Reward Questionnaire (BMRQ [36]) will be used to assess trait-level engagement with music. At the beginning of the study a brief self-report of therapy expectations and satisfaction with the treatment will be assessed, to account for effects of expectations of music therapy (see Fig. 3).

#### 2.5.2 Randomisation, blinding, and treatment allocation

Participants will receive two conditions in a randomised order: a) music-therapy intervention, and b) control condition involving standard care. The randomisation sequence will be computer generated (allocation 1:1). Due to the nature of the intervention, the clinical setting, and since the music therapist providing the intervention is one of the researchers, blinding of the researchers and assessors is not possible. However, the data analyses will be carried out blinded to the treatment order i.e. adding a round of pseudonymisation and nondescript labelling of experimental versus control periods.

#### 2.5.3 Study procedures

The protocol for this study has been approved by the Medical Research Ethics Committee of Leiden University/University of the Hague/Delft University, protocol number: NL78853.058.22. The study registered under ClinicalTrials.gov identifier: NCT06291480, with preregistered hypotheses at the Open Science Framework (https://osf.io/dzq7b).

Participants will be recruited ideally within the first three days after being admitted to the Leo Polak Rehabilitation Centre in Amsterdam, after review of the referral and handover from the main hospital. Patients will be first approached by a practitioner not involved in the study, who will inform the patient about the research study and provide the recruitment materials. If the patient indicates interest, the researcher will provide the patient with more information and the participant information letter, and if they wish to participate, they will be asked to sign the informed consent form.

An assessor will administer a checklist of exclusion/inclusion criteria to screen patients eligible to enrol in the study. Clinical measures administered at screening will be the Mini Mental State Examination (screening for cognitive impairment) (MMSE [41]), the National Institute of Health Stroke Scale (stroke severity) (NIHSS; [56]), and the Medical Research Council Scale for muscle strength (severity paresis arm/hand) (MRC-muscle strength [40]. All team members performing this screening are NIHSS-certified (Ortiz & Sacco, 2007). Additional medical information, such as type of stroke, location, lesion type etc, will be extracted from the client's medical dossier and triage process.

The intervention condition will consist of a target of 9 sessions in total, administered over an average of 5 weeks, but may span from 2 to 8 weeks according to the patient's length of stay at the Leo Polak Stroke Rehabilitation Centre. All music therapy interventions will be delivered by a registered/board certified/licenced music therapist who has had advanced training in NMT.

The protocol aims for participants to receive a minimum of two sessions per week over the duration of the study enrolment. However, this schedule may not be possible in every case due to the participants’ length of stay. Participants who complete a minimum of 6 sessions will be included in the final analyses. The number of sessions each participant receives will be recorded and treated as a variable in the statistical analysis.

Assessments will be conducted at three time-points during the trial. The measures will be administered at baseline (TP1), between the control and intervention conditions (mid-trial or TP2), as well as at the end of the trial after both conditions are completed (TP3).

Patients in the control period (Care as Usual) will receive standard care procedures that include a medical check-up, and interactions with (or treatments from) social workers and allied health practitioners, which include psychologists, nurses, physical therapists, occupational therapists, and speech and language therapists. In other words, Care as Usual includes all indicated therapies (e.g. physical or occupational therapy) minus NMT, and ranges on average between 5 and 7.5 h per week in total, depending on the rehabilitation trajectory, clients’ self-reliance, disciplines involved, recovery speed rate, and other individual characteristics.

### 2.6 Statistical analysis

Descriptive statistics for all relevant variables, such as changes in motivation, upper limb function and/or attention/concentration skills, will be calculated (means, standard deviations, etc.). Assumptions of all statistical analyses will be checked, and appropriate measures will be taken in case of violations (e.g., transformations, nonparametric testing, or bootstrapping). Effect sizes will be calculated for each effect (e.g., Cohen's *d*). Statistical tests will be performed with an alpha level of p ≤ .05 to determine statistical significance of results. Because of the randomised design, possible bias introduced by missing data will be controlled for using an intention-to-treat (ITT) analysis performed on all subjects who complete the baseline measurements, to assess whether data is missing randomly. If so, these may be completed with imputed data. Given the relatively small sample size of the study, this allows for a per-protocol analysis to also be conducted on datasets with missing data points, and the results from the ITT analysis will be verified on subjects who completed the entire study protocol, including all measurements (e.g. Ref. [57]). If needed, data analysis procedures with more robustness for missing data will be utilised (i.e., multilevel models). The self-reported motivation for sessions attended for other interventions offered at the rehabilitation centre will be recorded and compared between subjects as well as between sequences (AB/BA). Carry-over effects due to the cross-over design will be assessed by examining a treatment-by-period interaction. A significant difference between the two intervention or control phases of the AB/BA sequence, will result in significant treatment-by-period interaction indicating a carry-over effect (Lim & In, 2021). In case a significant carry-over effect is found, we will control for this in the analysis.

Other aspects that may be used as covariates in post-hoc analyses to interpret results at the group level are individual differences in diagnostic intake measures, age, and qualitative clinical observations or situations that may affect motivational states.

The primary analysis concerns the change in motivation scores (BMQ-S) between the control and intervention period, controlling for the order of conditions. Additionally, a correlation between changes in motivation with changes in functional outcome measures (motor, cognition, mood) will be calculated. For our secondary analysis, changes in functional measures will be compared between control and intervention periods to assess effectiveness of NMT. As exploratory analyses, correlations between functional improvements and possible predictors such as musical engagement, optimism, personality traits and expectations of NMT will be calculated. Finally, self-reported motivation for other rehabilitation specialisms will be compared between the intervention and control period. Participants will be included if they have completed at least 6 sessions of Music Therapy.

## 3 Discussion

The results of this research project can possibly have implications for practice, research and policy development. Music therapy is a discipline that is gaining acceptance and recognition, with a growing body of rigorous evidence for its use with people with acquired brain injury [20]. Although research supports music interventions for motor outcomes, more research is needed on specific interventions (such as NMT) to guide clinical practice and improve understanding of mechanisms and optimal dosage. Furthermore, the effects of music interventions to improve mood and motivational aspects are widely reported anecdotally, but need a stronger empirical basis. This study aims to inform the (health) practitioners on how and why music therapy and music-based interventions can be implemented as a primary or complementary approach to stroke rehabilitation. The methods described might potentially help music therapy clinicians find more accessible ways to use music therapy inspired by neuroscience, with focus on the empowering and motivating uses of music [9].

In terms of research, this relatively small-sized study can show that the field is ready for more studies that can conceptualise, measure and test a priori hypotheses in order to promote a more nuanced and mechanistic understanding of how (including mediators) and for whom (taking into account possible moderators) music therapy interventions are most effective, as also argued by Rodwin et al. [58].

10.13039/100018696Health care organisations, funding agencies and review panels that are less familiar with ‘non-traditional’ approaches, such as music therapy, might benefit from incorporating music therapy in future policies, regarding it as a useful and evidence-based rehabilitation discipline for stroke survivors, using experimental and theoretical knowledge on the effectiveness of music with clients with an acquired brain injury [20].

### 3.1 Underlying data

No data is associated with this article.

## Reporting guidelines

The Standard Protocol Items: Recommendations for Interventional Trials (SPIRIT) guidelines have been followed and a SPIRIT checklist can be found in the supplementary material S1.

## Grant information

The first author is suported by the Amstelring Rehabilitation Centres and Nursing Homes (Charitable Organisation in Amsterdam region, the Netherlands).

## CRediT authorship contribution statement

**Theo Dimitriadis:** Writing – original draft, Resources, Project administration, Methodology, Investigation, Funding acquisition, Conceptualization. **Mohammed A. Mudarris:** Writing – original draft, Methodology, Conceptualization. **Dieuwke S. Veldhuijzen:** Writing – review & editing, Project administration, Methodology. **Andrea W.M. Evers:** Writing – review & editing, Visualization, Conceptualization. **Wendy L. Magee:** Writing – review & editing, Supervision. **Rebecca S. Schaefer:** Writing – review & editing, Writing – original draft, Resources, Project administration, Methodology, Supervision, Conceptualization.

## Declaration of competing interest

The authors declare that they have no known competing financial interests or personal relationships that could have appeared to influence the work reported in this paper.

## Appendix A Supplementary data

Multimedia component 1 Additional reporting SPIRIT checklist

Multimedia component 2 SPIRIT checklist

## References

[1] Lee S., Lee K., Song C. (2018). Gait training with bilateral rhythmic auditory stimulation in stroke patients: a randomised controlled trial. Brain Sci..

[2] Leonardi S., Cacciola A., De Luca R., Aragona B., Andronaco V., Milardi D., Bramanti P., Calabrò R.S. (2018). The role of music therapy in rehabilitation: improving aphasia and beyond. Int. J. Neurosci..

[3] Tong Y., Forreider B., Sun X., Geng X., Zhang W., Du H., Ding Y. (2015). Music-supported therapy (MST) in improving post-stroke patients' upper-limb motor function: a randomised controlled pilot study. Neurol. Res..

[4] Rodriguez-Fornells A., Rojo N., Amengual J.L., Ripollés P., Altenmüller E., Münte T.F. (2012). The involvement of audio-motor coupling in the music-supported therapy applied to stroke patients. Ann. N. Y. Acad. Sci..

[5] Miller J.E., Carlson L.A., McAuley J.D. (2013). When what you hear influences when you see: listening to an auditory rhythm influences the temporal allocation of visual attention. Psychol. Sci..

[6] Schaefer R.S. (2014). Auditory rhythmic cueing in movement rehabilitation: findings and possible mechanisms. Phil. Trans. Roy. Soc. Lond. B Biol. Sci..

[7] Schneider S., Schönle P.W., Altenmüller E., Münte T.F. (2007). Using musical instruments to improve motor skill recovery following a stroke. J. Neurol..

[8] Thaut M.H., Hoemberg V. (2014). Handbook of Neurologic Music Therapy.

[9] Dimitriadis T., Della Porta D., Perschl J., Evers A.W.M., Magee W.L., Schaefer R.S. (2023).

[10] Jung H.Y., Lee S.H. (2017). Stroke Revisited: Diagnosis and Treatment of Ischemic Stroke.

[11] Kwakkel G., Kollen B.J., van der Grond J., Prevo A.J. (2003). Probability of regaining dexterity in the flaccid upper limb: impact of severity of paresis and time since onset in acute stroke. Stroke.

[12] Barker-Collo S.L., Feigin V.L., Lawes C.M., Parag V., Senior H., Rodgers A. (2009). Reducing attention deficits after stroke using attention process training: a randomised controlled trial. Stroke.

[13] Olgiati E., Russell C., Soto D., Malhotra P. (2016). Motivation and attention following hemispheric stroke. Prog. Brain Res..

[14] Grau-Sánchez J., Münte T.F., Altenmüller E., Duarte E., Rodríguez-Fornells A. (2020). Potential benefits of music playing in stroke upper limb motor rehabilitation. Neurosci. Biobehav. Rev..

[15] Veldkamp R., Goetschalckx M., Hulst H.E., Nieuwboer A., Grieten K., Baert I., Leone C., Moumdjian L., Feys P. (2021). Cognitive-motor interference in individuals with a neurologic disorder: a systematic review of neural correlates. Cognitive and behavioural Neurology : official journal of the Society for Behavioral and Cognitive Neurology.

[16] Verrienti G., Raccagni C., Lombardozzi G., De Bartolo D., Iosa M. (2023). Motivation as a measurable outcome in stroke rehabilitation: a systematic review of the literature. Int. J. Environ. Res. Publ. Health.

[17] Terry P.C., Karageorghis C.I., Curran M.L., Martin O.V., Parsons-Smith R.L. (2020). Effects of music in exercise and sport: a meta-analytic review. Psychol. Bull..

[18] De Witte M., Spruit A., van Hooren S., Moonen X., Stams G.J. (2020). Effects of music interventions on stress-related outcomes: a systematic review and two meta-analyses. Health Psychol. Rev..

[19] Finn S., Fancourt D. (2018). The biological impact of listening to music in clinical and nonclinical settings: a systematic review. Prog. Brain Res..

[20] Magee W.L., Clark I., Tamplin J., Bradt J. (2017). Music interventions for acquired brain injury. Cochrane Database Syst. Rev..

[21] Ripollés P., Rojo N., Grau-Sánchez J., Amengual J.L., Càmara E., Marco-Pallarés J., Juncadella M., Vaquero L., Rubio F., Duarte E., Garrido C., Altenmüller E., Münte T.F., Rodríguez-Fornells A. (2016). Music supported therapy promotes motor plasticity in individuals with chronic stroke. Brain Imaging and Behavior.

[22] Burger B., Thompson M.R., Luck G., Saarikallio S., Toiviainen P. (2013). Influences of rhythm-and timbre-related musical features on characteristics of music-induced movement. Front. Psychol..

[23] Baltazar M., Västfjäll D., Asutay E., Koppel L., Saarikallio S. (2019). Is it me or the music? Stress reduction and the role of regulation strategies and music. Music & Science.

[24] Kim S.Y., Kim J.M., Stewart R., Kang H.J., Kim S.W., Shin I.S., Park M.S., Cho K.H., Yoon J.S. (2013). Influences of personality traits on quality of life after stroke. Eur. Neurol..

[25] Dwan T., Ownsworth T. (2019). The Big Five personality factors and psychological well-being following stroke: a systematic review. Disabil. Rehabil..

[26] Oddy M., Cattran C., Wood R. (2008). The development of a measure of motivational changes following acquired brain injury. J. Clin. Exp. Neuropsychol..

[27] Gold C., Mössler K., Grocke D., Heldal T.O., Tjemsland L., Aarre T., Aarø L.E., Rittmannsberger H., Stige B., Assmus J., Rolvsjord R. (2013). Individual music therapy for mental health care clients with low therapy motivation: multicentre randomised controlled trial. Psychother. Psychosom..

[28] Ross S., Cidambi I., Dermatis H., Weinstein J., Ziedonis D., Roth S., Galanter M. (2008). Music therapy: a novel motivational approach for dually diagnosed patients. J. Addict. Dis..

[29] Mas-Herrero E, Dagher A, Farrés-Franch M, Zatorre RJ. Unraveling the temporal dynamics of reward signals in music-induced pleasure with TMS. Journal of Neuroscience. 2021 Apr 28;41(17):3889-99. 10.1523/JNEUROSCI.0727-20.2020. PMC8084325 33782048

[30] Gold B.P., Mas-Herrero E., Zeighami Y., Benovoy M., Dagher A., Zatorre R.J. (2019). Musical reward prediction errors engage the nucleus accumbens and motivate learning. Proc. Natl. Acad. Sci. USA.

[31] Belfi A.M., Loui P. (2020). Musical anhedonia and rewards of music listening: current advances and a proposed model. Ann. N. Y. Acad. Sci..

[32] Oyake K., Suzuki M., Otaka Y., Tanaka S. (2020). Motivational strategies for stroke rehabilitation: a descriptive cross-sectional study. Front. Neurol..

[33] Smelt A.F., van der Weele G.M., Blom J.W., Gussekloo J., Assendelft W.J. (2010). How usual is usual care in pragmatic intervention studies in primary care? An overview of recent trials. Br. J. Gen. Pract. : J. Roy. Coll. Gen. Pract..

[34] Turner K.M., Huntley A., Yardley T., Dawson S., Dawson S. (2024). Defining usual care comparators when designing pragmatic trials of complex health interventions: a methodology review. Trials.

[35] Grau-Sánchez J., Duarte E., Ramos-Escobar N., Sierpowska J., Rueda N., Redón S., Veciana de las Heras M., Pedro J., Särkämö T., Rodríguez-Fornells A. (2018). Music-supported therapy in the rehabilitation of subacute stroke patients: a randomized controlled trial. Ann. N. Y. Acad. Sci..

[36] Mas-Herrero E., Marco-Pallarés J., Lorenzo-Seva U., Zatorre R.J., Rodríguez-Fornells A. (2013). Individual differences in music reward experiences. Music Perception.

[37] Lyle R.C. (1981). A performance test for assessment of upper limb function in physical rehabilitation treatment and research. International journal of rehabilitation research. Internationale Zeitschrift für Rehabilitationsforschung. Revue internationale de recherches de réadaptation.

[38] Raglio A., Zaliani A., Baiardi P., Bossi D., Sguazzin C., Capodaglio E., Imbriani M. (2017). Active music therapy approach for stroke patients in the post-acute rehabilitation. Neurol. Sci..

[39] Fujioka T., Dawson D.R., Wright R., Honjo K., Chen J.L., Chen J.J., Ross B. (2018). The effects of music‐supported therapy on motor, cognitive, and psychosocial functions in chronic stroke. Ann. N. Y. Acad. Sci..

[40] Kleyweg R.P., van der Meché F.G., Schmitz P.I. (1991). Interobserver agreement in the assessment of muscle strength and functional abilities in Guillain-Barré syndrome. Muscle Nerve.

[41] Folstein M.F., Folstein S.E., McHugh P.R. (1975). "Mini-mental state". A practical method for grading the cognitive state of patients for the clinician. J. Psychiatr. Res..

[42] Altenmüller E., Marco-Pallares J., Münte T.F., Schneider S. (2009). Neural reorganisation underlies improvement in stroke-induced motor dysfunction by music-supported therapy. Ann. N. Y. Acad. Sci..

[43] Ramirez-Melendez R. (2023).

[44] van Alphen R., Stams G.J.J.M., Hakvoort L. (2019). Musical attention control training for psychotic psychiatric patients: an experimental pilot study in a forensic psychiatric hospital. Front. Neurosci..

[45] Street A.J., Magee W.L., Odell-Miller H., Bateman A., Fachner J.C. (2015). Home-based neurologic music therapy for upper limb rehabilitation with stroke patients at community rehabilitation stage-a feasibility study protocol. Front. Hum. Neurosci..

[46] Street A., Fachner J., Magee W. (2019). Upper limb rehabilitation in chronic stroke using neurologic music therapy: two contrasting case studies to inform on treatment delivery and patient suitability. Nord. J. Music Ther..

[47] Jones M.C. (2018).

[48] Stone S.P., Ali B., Auberleek I., Thompsell A., Young A. (1994 Sep-Oct). The Barthel index in clinical practice: use on a rehabilitation ward for elderly people. J R Coll Physicians Lond.

[49] Wechsler, D. WAIS-IV: Wechsler Adult Intelligence Scale–Fourth Edition, 4th ed. San Antonio: Pearson, 2008.

[50] Fine E., Delis D., Kreutzer J.S., DeLuca J., Caplan B. (2011). Encyclopedia of Clinical Neuropsychology.

[51] Golicki D., Niewada M., Buczek J. (2015). Validity of EQ-5D-5L in stroke. Qual Life Res.

[52] Lovibond S.H., Lovibond P.F. (1995). Manual for the Depression Anxiety Stress Scales.

[53] Stern R.A., Arruda J.E., Hooper C.R., Wolfner G.D., Morey C.E. (Jan. 1997). Visual analogue mood scales to measure internal mood state in neurologically impaired patients: Description and initial validity evidence. Aphasiology.

[54] Denissen J.J.A., Geenen R., Soto C.J., John O.P., van Aken M.A.G. (May 2020). The big five inventory–2: Replication of psychometric properties in a Dutch adaptation and first evidence for the discriminant predictive validity of the facet scales. J. Pers. Assess..

[55] Scheier M.F., Carver C.S., Bridges M.W. (Dec. 1994). Distinguishing optimism from neuroticism (and trait anxiety, self-mastery, and self-esteem): a reevaluation of the Life Orientation Test. J. Pers. Soc. Psychol..

[56] Ortiz G.A., Sacco R.L. (2014). Wiley StatsRef: Statistics Reference Online.

[57] Siponkoski S.T., Martínez-Molina N., Kuusela L., Laitinen S., Holma M., Ahlfors M., Jordan-Kilkki P., Ala-Kauhaluoma K., Melkas S., Pekkola J., Rodriguez-Fornells A., Laine M., Ylinen A., Rantanen P., Koskinen S., Lipsanen J., Särkämö T. (2020). Music therapy enhances executive functions and prefrontal structural neuroplasticity after traumatic brain injury: evidence from a randomized controlled trial. J. Neurotrauma.

[58] Rodwin A.H., Shimizu R., Travis R. (2023). A systematic review of music-based interventions to improve treatment engagement and mental health outcomes for adolescents and young adults. Child Adolesc Soc Work J.

