# Supplementary material for: Music therapy with adults in the subacute phase after stroke: A study protocol
Source: Contemp Clin Trials Commun. 2024 Jul 25;41:101340. doi: 10.1016/j.conctc.2024.101340 (PMC11345398; doi:10.1016/j.conctc.2024.101340)
Supplement: Multimedia component 1 [file mmc1.docx]

**Study status***

The inclusion of the subjects started in January 2023, with the goal of completing inclusions of participants in April 2024. Publication of the results is anticipated to take place between the second half of 2024 and the first half of 2025.

**Dissemination**

As this scientific research involves human subjects, all results will be disclosed unreservedly, i.e. regardless of confirmation or disconfirmation of the hypotheses. All parties concerned will justify their actions in this regard. Both positive and negative trial results will be disclosed and submitted for publication to peer-reviewed scientific journals. In case that the journal does not consider negative results for publication, other ways of disclosing research results will be sought, such as trial registers (clinicaltrials.gov), and databases (Yamey, 1999; CCMO, 2023).

**Ethical considerations***

*Withdrawal of individual subjects*

Subjects can leave the study at any time for any reason if they wish to do so, without any consequences. The investigator can decide to withdraw a subject from the study for urgent medical reasons, or other factors that pertain to the inclusion criteria.

*Replacement of individual subjects after withdrawal*

Participants who voluntarily withdraw from the study prematurely, or who are withdrawn at the discretion of the researcher for technical or medical reasons will be replaced by recruiting additional participants (replacement arrangement), such that the sample size meets the minimum of study completers. All data from participants that ask to be withdrawn from the study will be excluded from analysis.

*Premature termination of the study*

We do not expect premature termination to be necessary, however, the study may be prematurely terminated if it proves impossible to recruit enough eligible participants, or if the majority of participants were not able to complete the protocols or engage in the intervention.

*Regulation statement*

The study will be conducted according to the principles of the Declaration of Helsinki (2 October, 2013), in accordance with the Medical Research Involving Human Subjects Act (WMO) in The Netherlands and the General Data Protection Regulation (GDPR), and has been granted formal approval by the medical ethical committee of Leiden, the Hague and Delft Universities, registration number NL78853.058.22- P22.014. All researchers involved in these projects (will) hold the necessary legal qualifications in order to conduct clinical research with human subjects in the Netherlands.

Back matter

**Data availability***

A data management plan (DMP) has been developed prior to the writing of this protocol and is approved by the data management officer of the department of Health, Medical and Neuropsychology of Leiden University.

Personal identification codes will be used to link to participant data. The file containing the encryption key between participant’s number and personal data (e.g., name) will be managed by researchers and the data manager, but otherwise locked for access from others. If a participant has withdrawn from the study, their data will be stored, but will not be shared or published. Any data that is published or shared outside the research group will not be traceable to personal information.

*Underlying data**

No data is associated with this article.

**Competing interests***

There are no competing interests for the realisation of this study.

CCMO (Central Committee on Research Involving Human Studies) (2023-08-28) CCMO Statement on publication policy. CCMO. URL: <https://english.ccmo.nl/publications/publications/2002/03/01/ccmo-statement-on-publication-policy>.

G. Yamey, Scientists who do not publish trial results are “unethical”, BMJ Br. Med. J. (Clin. Res. Ed.) 319 (7215) (1999) 939.
